# Transcranial direct current stimulation combined with language-cognitive training improves language and cognitive ability in children with language delay

**DOI:** 10.3389/fneur.2024.1412959

**Published:** 2024-07-12

**Authors:** Huichang Zhou, Yunxian Xu, Lishan Chen, Jiajian Yuan, Zhiyong Guan, Peng Liang

**Affiliations:** The First People’s Hospital of Foshan, Guangdong, China

**Keywords:** language delay, transcranial direct current stimulation (tDCS), language-cognitive training, home training, Sign-signification relations (S-S) test

## Abstract

**Introduction:**

Language delay cannot be ignored, and there is an urgent need to determine therapies that elicit better results in a short period. However, whether transcranial direct current stimulation (tDCS) alone or in combination with other therapies can promote recovery of language and cognitive function in children with language delay remains unknown. This study aims to explore the effects of tDCS combined with language-cognitive training and home-based rehabilitation on language and cognitive ability in children with language delay.

**Methods:**

Children with language delay who visited the Department of rehabilitation medicine or the pediatric outpatient clinic of the First People’s Hospital of Foshan from January 2019 to December 2021, totaling 190 in number, were included and randomly divided into 4 groups, i.e., the family guidance group, the tDCS group, the language-cognitive training group, and the comprehensive training group. The family guidance group (47 cases) received home training. The tDCS group (46 cases) received home training and tDCS treatment. The language- cognitive training group (49 cases) adopted home training and language-cognitive training. The comprehensive training group (48 cases) took home training, language-cognitive training, and tDCS treatment. All groups received training 5 times a week for 4 weeks. The Sign-significant relations (S-S) test was applied to evaluate the language comprehension, language expression, basic learning ability, and attitude of communication of the children.

**Results:**

The language-cognitive training group and the comprehensive training group showed improvement after treatment (*p* < 0.05) regarding basic learning ability. The communication attitude of the four groups improved after intervention (*p* < 0.05). Particularly, the comprehensive training group had maximum improvement after intervention. No serious adverse reactions such as epilepsy, headache, and behavioral abnormalities were found.

**Conclusion:**

tDCS combined with language-cognitive training and home training can improve language and cognitive ability in children with language delay.

## 1 Introduction

Language delay refers to the condition whereby a child’s language development does not reach an appropriate level for their age. The prevalence rate of preschool children with language delay is approximately 7.4% in the United States and at least 5 ~ 12% in China (1, 2). Delay in children’s language affects their ability to adapt to society and increases the incidence of psychological and behavioral problems, such as attention deficits and learning difficulties (3). Without prompt treatment, 40 to 60% of children may continue to have speech and language difficulties, with a higher risk of social, emotional, behavioral, and cognitive problems later in life (1). Long-term cohort studies have found that children with late language maturity are more likely to have narrative and reading-related learning difficulties by the end of primary school, even if they catch up with normally developing children at ages 4 to 7 (4). Numerous studies have proved that language delay cannot be ignored, and early language intervention achieves better outcomes.

Children’s development is related to their environment and interactions (5). Tosh assumes that family training can improve the speech and language skills of children with language difficulties (6). Zhao et al. believe that early family training can improve their language level and communication skills (7). Therefore, family training is important for children’s language development (8). However, implementing home training is not easy, and it requires parents to have a good level of expertise and compliance. Due to limitations in parents’ compliance and children’s cooperation, family training is rarely used in the treatment of language delay.

Accumulating evidence suggests that speech/language therapy and cognitive training can promote the development of children’s language and cognitive functions through systematic training of speech, memory, hearing, and expression skills (9). By direct intervention of language and indirect intervention of factors that influence language in children through comprehensive intervention, improvement in language and cognition in children can be achieved, which is helpful during early treatment. However, only a small number of families can receive long-term treatment. Whether the effects of such treatment can be evident during a relatively short period remains unknown. Hence, there is an urgent need to determine therapies that elicit better results in a short period.

Non-invasive brain stimulation (NIBS) methods have been widely utilized in research settings to manipulate and understand the functioning of the human brain. The use of NIBS for the recovery of language and cognitive functions has been well documented (10). Qiu et al. found that combining transcranial magnetic stimulation (TMS), an NIBS technique, and language training can effectively improve linguistic competence, action ability, and mouth movement in children with language delay (11). Transcranial direct current stimulation (tDCS) is another NIBS technique that uses weak currents to regulate cortical excitability (12). tDCS with concurrent computerized cognitive training could enhance cognitive function in stroke patients (13). It was also found that the combination of tDCS and speech-language therapy or performing a writing task could improve performance of language and cognitive tasks for stroke patients (14, 15). However, whether tDCS alone or in combination with other therapies can promote recovery of language and cognitive function in children with language delay remains unknown.

In the current study, we investigated the effects of tDCS, home training, language-cognitive training, and the combination of these therapies on language and cognitive function in children with language delay.

## 2 Materials and methods

### 2.1 Study design

This study was a prospective, randomized, controlled, and assessor-blind trial. The Research Ethics Committee of the First People’s Hospital of Foshan approved this study (Approval Number: L [2017] No. 6), and written informed consent was obtained from the legal guardian of each participant.

Each patient was randomly assigned to one of four groups: the family guidance group, the tDCS group, the language-cognitive training group, and the comprehensive training group. A qualified assessor conducted assessments and administered the Sign-signification relations (S-S) test. The assessors were blinded to the group assignments. Children were evaluated at baseline and 4 weeks after randomization (that is, immediately after the 4-week intervention).

### 2.2 Participants

Overall, 220 children with language delay who visited the Department of rehabilitation medicine or pediatric outpatient clinic of the First People’s Hospital of Foshan between January 2019 and December 2021 were included. According to the random number produced by Statistical Product and Service Solutions for Windows (release 22.0, SPSS), the children were divided into four groups in a 1:1:1:1 ratio.

### 2.3 Inclusion and exclusion criteria

The inclusion criteria comprised the following: (1) Children diagnosed with “language disorder” by the Diagnostic and Statistical Manual of Mental Disorders (5th Edition) (DSM-V) (16). (2) Determined as “language delay” by using the S-S test. (3) Age 2–6 years. (4) The visual, auditory, and pronunciation functions were normal. (5) Right-handed. (6) The guardians of the children signed the informed consent.

The exclusion criteria comprised the following: (1) Children with language delay caused by neurological diseases such as cerebral palsy, traumatic brain injury, and meningoencephalitis. (2) Children with speech disorders caused by mental and organic diseases such as autism, cleft lip and palate, hearing impairments, oral motor dysfunction, attention deficit hyperactivity disorder, familial language development delay, acquired aphasia due to epilepsy, specific language impairment, or mild deprivation of language environment (17). (3) Children with language barriers such as stuttering and cluttering. (4) Presence of contraindications of tDCS. (5) Children who cannot eat orally, such as those requiring a nasal feeding tube or gastrostomy. (6) Children with serious illnesses who cannot complete the trial. (7) The parent or guardian of the child is a professional in rehabilitation or child growth and development.

### 2.4 Interventions

The family guidance group received home training alone, the tDCS group received home training + tDCS treatment, the language-cognitive training group received home training + language-cognitive training, and the comprehensive training group received home training + language-cognitive training + tDCS treatment.

#### 2.4.1 Home training

The home training was set by the therapist providing oral guidance to the child’s guardians on how to train at home, with the guidance referring to the language-cognitive training content (see below) based on the result of the S-S test (18). Home training was performed 30 min per day, and 5 days per week, for 4 weeks. To ensure the consistency of home training, the child’s guardians were asked to complete a record form, noting the duration, content, and the child’s response for each training session. Regular feedback and discussion with the therapist were required, and the record form was handed over to the researcher at the end of the study.

#### 2.4.2 tDCS treatment

A Ruihai IS200 transcranial direct current stimulation instrument with an electrode size of 5 × 7 cm was used. The anode stimulated the temporal lobe centered on the left Wernicke’s area, and the cathode was placed on the right scapula (19). The current intensity was 1 mA, and the current density was 0.029 mA/cm^2^. The tDCS treatment was given for twenty minutes each time, once a day, five times a week, for a total of four weeks.

Left Wernicke’s area was located as follows (19): Measure the head with a tape from the inion to the nasion, and note the middle of this distance. Then, measure the distance from the left preauricular point to the right preauricular point, and mark the crosspoint of the two measurements. Measure 30% of the distance between the preauricular points from the crosspoint down the left hemisphere and mark it. Measure 10% of the distance between the inion and the nasion from the marked point to the back of the head. This point is the Wernicke’s area location.

#### 2.4.3 Language-cognitive training

The language-cognitive training included one-on-one language training, cognitive training, and social interaction training. The training was provided 30 min per day and 5 days per week for 4 weeks. Both the content of home training and language-cognitive training were formulated by the speech therapist according to the child’s language development stage after the first S-S assessment. The language-cognitive training was conducted one-on-one by the speech therapist, whereas the home training involves the introduction provided by the therapist to the child’s guardians on how to train the child at home, with the guidance content referring to the language-cognitive training content. The training principle is to carry out horizontal expansion within the same stage whereas vertically advancing to the next stage (20). For example, during the training process, for children who have a good understanding of the names of things, therapists expand the vocabulary within the noun level then expand horizontally to verbs and adjectives, and then vertically advance to the next stage, phrase training.

1. Training of gestural symbols: Gestural symbols use hand gestures as indicative symbols with certain meanings, expressing intentions and communicating non-verbally to others through these gestures. Compared with verbal symbols, especially adult language, gestural symbols have a more direct, vivid, and comprehensible relationship with objects they refer to, such as clapping hands together and placing them on one side of the face to indicate “sleeping.” Gestural symbols are easier for children to understand, master, and operate. They are suitable for training children who have not yet mastered the understanding and expression of verbal symbols, as well as children who can understand verbal symbols but are unable to express them.
2. Expansion of verbal symbols—vocabulary training: This stage of training is crucial in language development delay. The expression of verbal symbols (verbal expression) is based on the premise of sound symbols (verbal understanding) at this stage. The biggest difference between this stage and the gestural symbol stage is that the gestural symbol stage helps children understand the names of things through actions whereas this stage trains the child to respond appropriately with only sound symbols. The acquisition of the relationship between symbolic forms and indicated content at this stage can be carried out through a gradual progression from body language symbols to child language, and then to adult language.
3. Phrase training: For training words, the symbolic forms composed of several words are called phrases. This stage is suitable for children who can understand the elements that make up phrases, such as names of things, verbs, and adjectives. The training content starts from the corresponding two-word phrases, three-word phrases, the construction of language forms, the formation of concepts, and picture selection that combines with the language form at the sentence level (indicated content) for comprehension training and promoting the expression of verbal and written symbols.
4. Grammar training: That is, training the rules of forming sentences from words. For children to master language and communicate verbally, they must also master the grammatical system. Based on the ability to understand and express simple sentences, children were trained to learn complex sentences (reversible sentences, passive sentences).

#### 2.4.4 Comprehensive training

The children in the comprehensive training group received three treatments: language-cognitive training, tDCS, and home training. Each treatment was given once a day and five times a week for four weeks.

### 2.5 Evaluation

#### 2.5.1 Language and cognitive function

The language and cognitive function of each child before and after training were evaluated using the S-S test (21), which was developed by the National Rehabilitation Center in Japan. The Language Therapy Department of the China Rehabilitation Research Center (CRRC) revised it according to the language development patterns of Chinese children and the Chinese language system, creating the CRRC version of the S-S test. It included four parts: language comprehension, language expression, basic learning ability, and attitude of communication. Language comprehension ability typically refers to “listening comprehension.” Language expression ability pertains to the use of linguistic symbols, often referring to “what to say.” Basic learning ability involves visual and auditory discrimination, memory, and reproduction skills. Communication attitude refers to the willingness and engagement in daily communication.

This is currently one of the most commonly used testing tools in the diagnosis and rehabilitation evaluation of language disorders in China, suitable for children aged 1.5 to 6.5 years old (22). It divides the level of language development into five phases, with further subdivisions within each stage (as shown in Table 1). Comparing the evaluation results with the age level of healthy children can intuitively identify children with language delay and the gap between the expected age level and their actual age. If the results are below the expected age level, the corresponding items can be identified as abnormal. Moreover, it can guide the goals and content of children’s language training based on the assessment results.

**Table 1:** Phases and content meanings of S-S test.

Phase	Content	Stage	Corresponding age (year)
One: Pre-linguistic stage	Struggling to understand situations and events, capable of noticing things and the actions or sounds of others, and able to actively respond to external stimuli (such as movements); however, unable to comprehend specific things. For example, unable to differentiate between food and non-food items, and placing non-food items into the mouth as well.	1: Difficulty in understanding things	
Two: Basic concepts of things	Can understand the interrelationships of things that appear or exist in daily life, for example: when the father takes out a cigarette, the child with special needs hands over a match, but has difficulty in understanding and using symbols.	2–1: Functional operations	
		2–2: Matching items	
		2–3: Selecting items	
Three: The symbols of things	Can distinguish between symbols and the items they represent (establishing the correspondence between symbols and objects), and can comprehend the meaning of symbols, such as gestures, baby talk, onomatopoeia, and mimetic words. For example: performing the action of putting the telephone receiver to the ear, or selecting a telephone toy based on sound.	3–1: Gestural symbols (related symbols)	
		3–2: Verbal symbols: Infant language (related symbols)/Adult language (arbitrary symbols)	1.5~
Four: Phrase, main sentence constituents	Can understand phrases made up of words in a child language manner, at this time it is necessary to observe the child’s understanding of Chinese phrases from a developmental perspective. For example: he can understand “telephone,” “girl,” “dog watching person,” but cannot say them.	4–1: Two-word phrase	2~
		4–2: Three-word sentence	2.5~
Five: Lexical phrase, grammatical rules	Can understand at the same level as an adult. Phrases (sentences) are formed according to grammatical rules, and can understand both simple and complex sentences. For example: “A person is watching TV,” “After finishing their meal, a person watches an interesting TV program.”	5–1: Word order	3.5~
		5–2: Passive voice	5 ~ 6.5

The examination order and content of the S-S test are as follows: The examination of basic learning ability includes items such as throwing a small ball, delayed response, shape discrimination, block building, tracing lines, imitation, and auditory memory span. The examination of the relationship between symbols and indicated content, assessing language comprehension and language expression levels, includes functional operations, matching and selection, gestural symbols and verbal symbols, two-word and three-word sentences, and checks on word order and passive voice sentence structures. The examination of daily life communication attitudes includes attention to others’ actions, eye contact, response to others’ instructions, greetings, reactions to being greeted, expressing intentions to others, expression of emotional fluctuations, question-and-answer relationships, and characteristic speech.

The scoring of the S-S test is based on the child’s performance in the aforementioned examination items. Each stage and level has specific content to be assessed, and the evaluator determines the developmental stage of the child based on their responses and abilities.

#### 2.5.2 Comprehensive score

In order to comprehensively evaluate the level of each child’s language ability, we refer to the KSAOs (knowledge, skills, abilities and other characteristics) (23), which is a common model application principle used to evaluate a person’s overall ability, and then obtain the following calculation formula. By converting these scores into a spider web diagram, the larger the area enclosed by the spider web, the higher the overall ability is indicated. The score of each of the four parts of the S-S test was normalized with the formula: Y = (X-Xmin) / (Xmax–Xmin). After normalization, a comprehensive score was calculated with the formula: Comprehensive score = (a*b + b*c + c*d + d*a)/2, in which normalized language comprehension score = a, normalized language expression score = b, normalized basic learning ability score = c, and normalized attitude of communication score = d.

#### 2.5.3 Efficacy classification

The results of the S-S test should be compared with the actual age of the child; a result lower than the actual age indicates an abnormal status (21). Here, based on whether post-treatment S-S scores match children’s actual age, the efficacy of treatment was classified into four groups: no effect, mild effect, moderate effect, and remarkable effect (20): A score on the S-S test that was in line with the child’s actual age was considered “remarkable effect”; an increase in the S-S test score of more than one phase was considered “moderate effect”; an increase in the S-S method score that did not reach one phase was considered “mild effect”; and no improvement or a decrease in the S-S method score was considered “no effect.” The total effective rate was calculated as the sum of the remarkable effect rate, moderate effect rate, and mild rate.

#### 2.5.4 Adverse reactions

We observed whether the children in each group had adverse reactions such as epilepsy, headache, and abnormal behavior during training and treatment.

### 2.6 Statistical methods

SPSS (version 22.0) was used for statistical processing of data. One-way analysis of variance was used to compare measurement data between the groups, the paired t-test was used for comparison before and after treatment, and the Kruskal–Wallis H test was used to compare ranked data between the groups as the results of the S-S test were ordinal data. The chi-square test was conducted for the comparisons of age and therapeutic efficacy across the four groups. The statistical significance level was set at *p* < 0.05, and a two-tailed test was used.

## 3 Results

**Figure 1:**
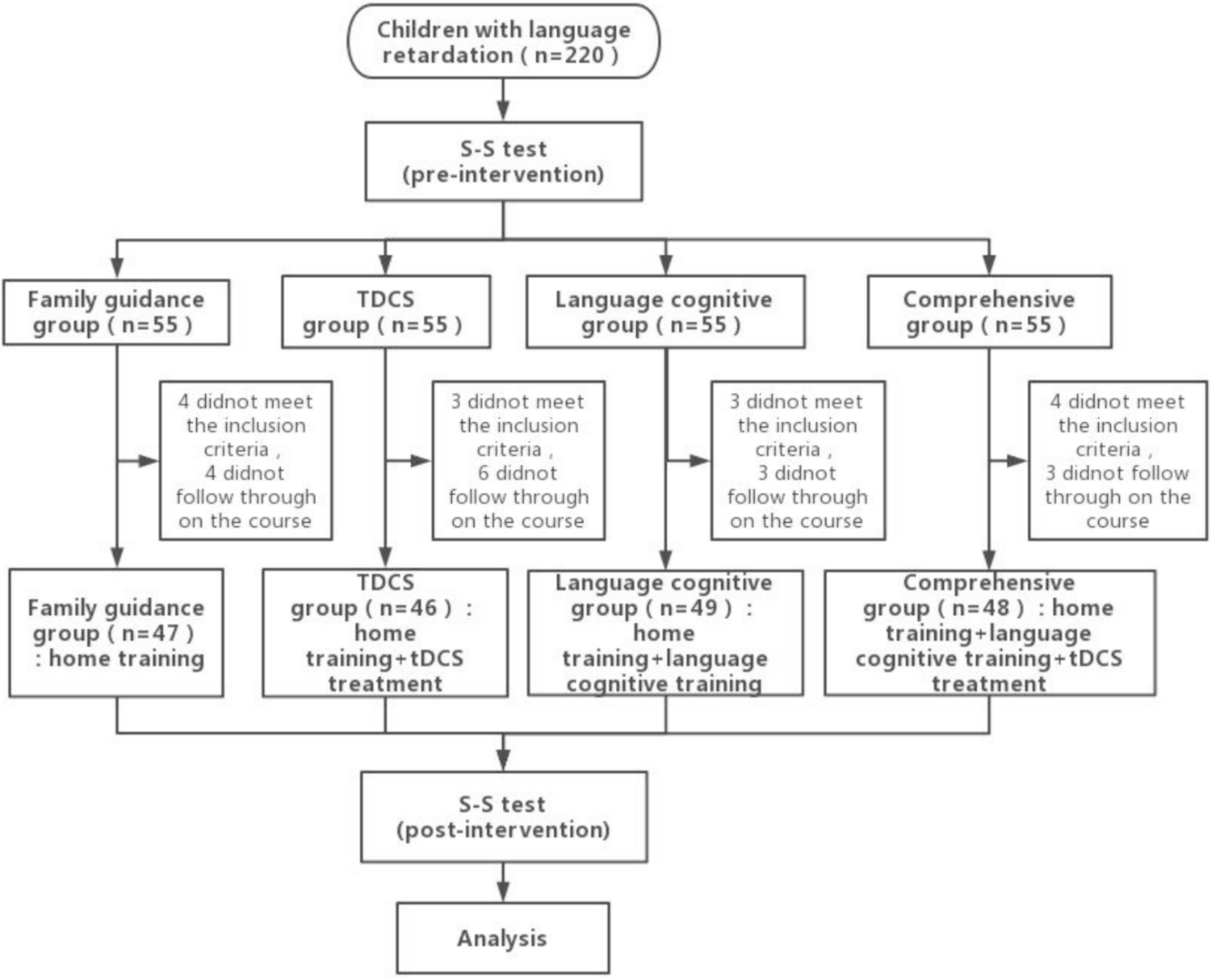
Study flow diagram.

Fifty-five children were assigned to each group. After assignment, in the family guidance group, 4 patients did not meet the inclusion criteria after the S-S test, and 4 could not complete the treatment course; 47 cases were finally included, including 24 males and 23 females, aged 2.2 ~ 5.8 (3.55 ± 0.88) years old. In the tDCS group, 3 cases did not meet the inclusion criteria after the S-S test, 6 could not persist in completing the treatment course, and 46 cases were finally included, including 22 males and 24 females, aged 2.5 ~ 5.2 (3.67 ± 0.58) years old. In the language-cognitive training group, 3 patients did not meet the inclusion criteria after evaluation, and 3 could not complete the treatment course; 49 cases were finally included, including 25 males and 24 females, aged 2.5 ~ 5.3 (3.56 ± 0.82) years old. In the comprehensive training group, 4 patients did not meet the inclusion criteria after being evaluated by the S-S test, and 3 patients could not complete the treatment course; 48 cases were finally included, including 23 males and 25 females, aged 2.2 ~ 5.9 (3.88 ± 0.87) years old. There were no significant differences in age or sex among the four groups (*p* > 0.05) (Table 2; Figure 1).

**Table 2:** Baseline participant characteristics.

Group	Case	Male	Female	Age (y)	Mean ± SD (y)
Family guidance	47	24	23	2.2–5.8	3.55 ± 0.88
tDCS	46	22	24	2.5–5.2	3.67 ± 0.58
Language-cognitive training	49	25	24	2.5–5.3	3.56 ± 0.82
Comprehensive training	48	23	25	2.2–5.9	3.88 ± 0.87

tDCS, transcranial direct current stimulation.

### 3.1 Language and cognitive ability

**Figure 2:**
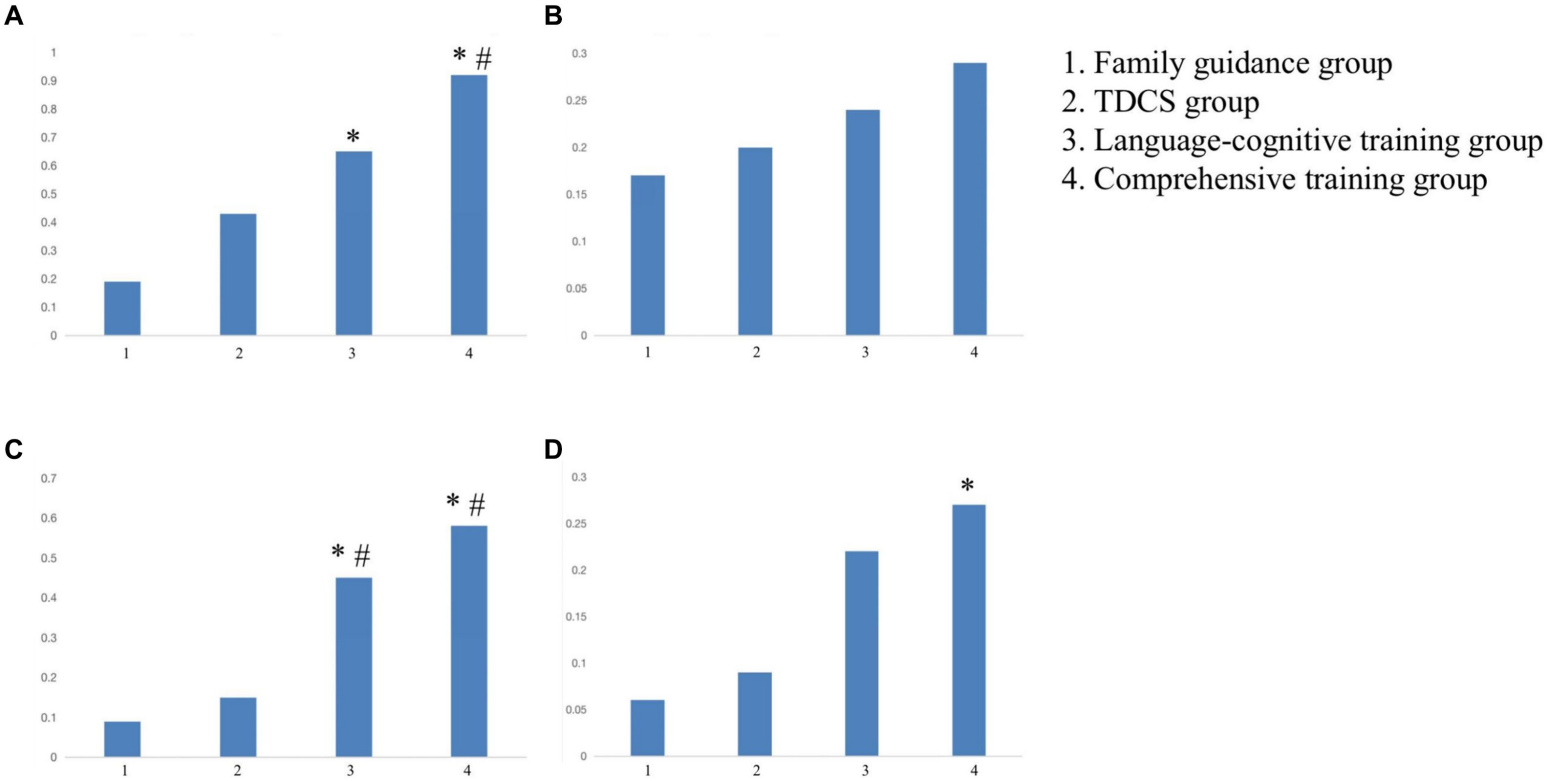
The difference of pre- and post-intervention scores of the S-S test in the four groups. **(A)** Comprehension ability. **(B)** Expressive ability. **(C)** Basic learning ability. **(D)** Communication attitude. *Compared with the family guidance group, *p* < 0.05. #Compared with the tDCS group, *p* < 0.05. tDCS, transcranial direct current stimulation.

There was no statistically significant difference in language comprehension, language expression, basic learning ability, and attitude of communication scores among the four groups before training (*p* > 0.05). The language-cognitive training group and comprehensive training group were significantly higher than the family guidance group and tDCS group in the language comprehension ability and basic learning ability of the S-S test (*p* < 0.05). Except for the changes in the basic learning ability of the family guidance group, all four parts of the S-S test exhibited significant improvements (*p* > 0.05) in each of the four groups (Table 3; Figure 2).

**Table 3:** Pre- and post-intervention changes in the S-S test across the four groups (χ ± S).

Group	Case	Language comprehension		Language expression		Basic learning ability		Communication attitude	
		Pre-intervention	Post-intervention	Pre-intervention	Post-intervention	Pre-intervention	Post-intervention	Pre-intervention	Post-intervention
Family guidance	47	3.45 ± 1.84	3.64 ± 1.87^*^	1.32 ± 0.52	1.49 ± 0.75^*^	1.76 ± 0.72	2.04 ± 0.69	1.17 ± 0.39	1.23 ± 0.43^*^
tDCS	46	3.39 ± 1.73	3.83 ± 1.90^*^	1.30 ± 0.63	1.50 ± 0.78^*^	1.80 ± 0.72	1.96 ± 0.63^*^	1.17 ± 0.38	1.26 ± 0.44^*^
Language- cognitive training	49	3.33 ± 1.69	3.98 ± 1.81^*^	1.37 ± 0.78	1.61 ± 1.00^*^	1.67 ± 0.75	2.12 ± 0.86^*^	1.20 ± 0.41	1.43 ± 0.50^*^
Comprehensive training	48	3.83 ± 1.72	4.75 ± 1.98^*^	1.54 ± 0.92	1.83 ± 1.06^*^	1.46 ± 0.58	2.04 ± 0.71^*^	1.27 ± 0.45	1.54 ± 0.50^*^
*F*		0.820	3.171	1.060	1.468	1.395	0.426	0.625	4.511
*P*		0.484	0.026	0.368	0.225	0.265	0.734	0.600	0.004

^*^Compared with the score before training, *p* < 0.05. tDCS, transcranial direct current stimulation.

### 3.2 Comprehensive score

There were no significant differences in the pre-intervention comprehensive scores of the four groups (*p* > 0.05). After training, the comprehensive scores of all the four groups increased, and the comprehensive score of the comprehensive training group was the greatest, followed by tDCS group, language-cognitive training group, and family guidance group (the score decreased in this order). Further, this difference was statistically significant (*p* < 0.05) (see Table 4).

**Table 4:** Pre- and post-intervention changes in the comprehensive scores in the four groups (χ ± S).

Group	Case	Pre-intervention	Post-intervention	*t*	*P*
Family guidance	47	48.02 ± 7.19	53.26 ± 8.16	3.303	0.001
tDCS	46	48.12 ± 6.86	60.02 ± 7.84^a^	7.831	0.000
Language-cognitive training	49	47.98 ± 7.25	56.36 ± 6.98^ab^	5.709	0.000
Comprehensive training	48	48.11 ± 7.32	64.15 ± 7.88^abc^	10.224	0.000
*F*		0.005	28.700		
*P*		0.995	0.000		

^a^Compared with the family guidance group, *p* < 0.05. ^b^Compared with the tDCS group, *p* < 0.05. ^c^Compared with the language-cognitive training group, *p* < 0.05. tDCS, transcranial direct current stimulation.

### 3.3 Efficacy

**Figure 3:**
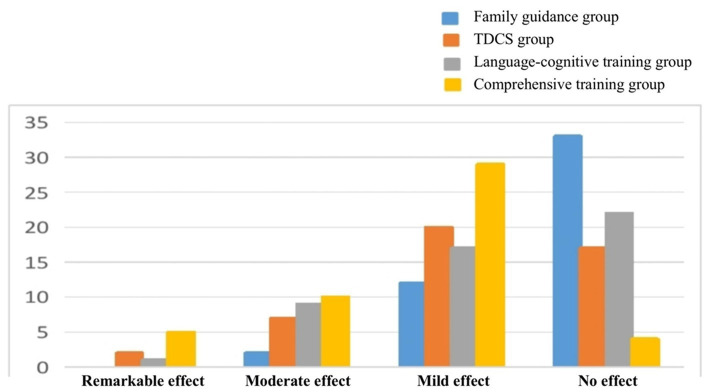
Effects of the four groups. tDCS, transcranial direct current stimulation.

The total effective rate of the comprehensive training group was higher than those of the other three groups, and the difference was statistically significant (*p* < 0.05). The total effective rate of the tDCS and language-cognitive training groups was higher than that of the family guidance group (*p* < 0.05). There was no statistically significant difference between tDCS and language-cognitive training group (*p* > 0.05) (see Table 5; Figure 3).

**Table 5:** Effects of the four groups.

Group	Case	Remarkable effect (%)	Moderate effect (%)	Mild effect (%)	No effect (%)	Total effective rate (%)
Family guidance	47	0 (0.00)	2 (4.26)	12 (25.53)	33 (46.81)	14 (29.79)
tDCS	46	2 (4.35)	7 (15.22)	20 (43.48)	17 (36.96)	29 (63.04)
Language-cognitive training	49	1 (2.04)	9 (18.37)	17 (34.69)	22 (44.90)	27 (55.10)
Comprehensive training	48	5 (10.42)	10 (20.83)	29 (60.42)	4 (8.33)	44 (91.67)
χ^2^						38.599
*P*						0.000

tDCS, transcranial direct current stimulation.

### 3.4 Safety

In this trial, no severe adverse reactions, such as epilepsy, headaches, or abnormal behavior were reported, and no skin burning, redness, or swelling was observed. Skin flushing occurred at the electrode application site after tDCS treatment and subsided after a few minutes. Five children (three in the comprehensive group and two in the tDCS group) reported discomfort at the beginning of the tDCS treatment, which resolved spontaneously after a few seconds.

## 4 Discussion

Firstly, this study found that simple family guidance alone could improve the functionality of children with language delay. Secondly, the study discovered that tDCS, or tDCS in combination with other therapies, could enhance the functionality of children with language delay. Thirdly, the study found that a brief period of rehabilitation intervention (4 weeks) could improve the functionality of children with language delay, which is shorter than the previously reported duration of 6 weeks to 3 months (24–26). These are novel findings not reported in prior research. Communication attitude significantly improved after home training, language-cognitive training, tDCS, and comprehensive training. Comprehensive training was more effective, and comprehension ability, communication attitude, comprehensive score, and total effective rate were significantly higher than those of the other three groups.

Early childhood is a critical period of language development. Language delay not only seriously affects children’s language comprehension and speech expression ability, but also affects children’s social adaptability and increases the incidence of psychological and behavioral problems, such as attention deficit and learning difficulties (27, 28), which can lead to deficits in cognitive abilities, including attention, executive abilities, memory, and so on (29). In clinical practice, children with language delay often experience problems such as attention deficits, memory impairment, and executive ability deficits. Cognitive abilities also play an important role in improving language skills, helping children establish appropriate communicative and social behaviors, and acquiring more information about the world through language (30). This study compared the intervention effects of home training, tDCS, language-cognitive training, and comprehensive training groups on children with language delay to investigate how cognitive abilities in language can be improved more effectively.

Studies have shown that early intervention and structured family activity programs positively impact brain development in children with global developmental delays, emphasizing the important role of parental involvement (31, 32). An increasing number of studies are advocating for the implementation of interventions for children with developmental delays in natural settings, with families being the natural setting to which children are exposed the most (33, 34). After the family guidance group receives home rehabilitation guidance, parents can conduct the treatment more suitably, and children can receive various rehabilitation training programs in a warm and familiar family environment, which can minimize their resistance to rehabilitation training. Furthermore, home training under guidance may be conducive to creating a friendly environment for children and promoting their language development. Our results showed that children’s communication attitudes in the family guidance group improved after treatment, which was consistent with the findings of the previous studies. When there is good interaction between caregivers and children, the children’s cognitive, social, and emotional development improves (35, 36).

Cognitive/language training is a commonly used clinical intervention for language delay. The training provides early language and cognitive stimulation for children to improve the function of the brain’s language and cognitive centers (37, 38). Language-cognitive training mainly stimulates sensory organs through listening, speaking, watching, reading, etc. It activates the language and cognitive function in the brain, helps functional reorganization of brain networks, promotes the development of children’s language and cognitive function, and improves their adaptability and communication attitude (11). Consistent with the previous studies (39), in this study the comprehensive scores, operational items, and communication attitudes of the language-cognitive training group improved compared with those before treatment, indicating that language-cognitive training has a positive effect on language delay and promotes language development in children in terms of cognitive and social development. However, the effect of language-cognitive training cannot be achieved in a short time in the previous studies (26, 39), leading to participation in long-term training in clinical practice, which can easily fail. Therefore, it is urgent to find a more efficient method.

tDCS is a promising non-invasive brain stimulation tool that can potentially improve brain function in adults and pediatric populations in cerebral palsy, epilepsy, autism spectrum disorders, childhood developmental disorders, attention-deficit/hyperactivity disorder (ADHD), and other pediatric diseases (40). In this study, the tDCS anode was placed on the left temporal lobe of the child to stimulate the Wernicke’s area, which is an important channel for language comprehension, so that the subsequent components of the language chain (e.g., language expression) also improve accordingly. In addition, Lefaucheur et al. (41) reported that tDCS stimulation is not highly localized and may produce nonspecific effects (such as attention and memory retrieval). Studies have also found that tDCS can modulate perfusion of the cortex and regulate brain metabolism, thereby optimizing brain function (42, 43), which supports the findings of this study. In the tDCS group, communication attitude significantly improved compared with that before treatment. However, it was not different from the language-cognitive training group (*p* > 0.05), indicating that tDCS can improve outcomes of language delay.

The results of this study showed that the language comprehension, language expression, basic learning ability, and attitude of communication of the comprehensive training group improved compared with those before treatment. Also, the language comprehension and attitude of comprehension and communication were higher than those of the other three groups, indicating that tDCS combined with language-cognitive training positively improves the comprehension and communication attitude of children with language delay and is better than only language-cognitive training or tDCS treatment. Lu et al. found that combining tDCS and working memory training enhanced individual cognitive function effectively (44). Similarly, in this study, tDCS treatment was administered together with conventional language training. The results support that such comprehensive treatment can superimpose the effects of language-cognitive training and tDCS training and thus can make greater progress for children with language delay.

Research indicates that 3 to 5 years old is the optimal age for treating children’s language disorders, and treatment at this stage can significantly reduce the long-term and short-term impacts caused by language disabilities (45, 46). Considering that some children’s language development delays may resolve on their own as they grow older, we established a family guidance group to eliminate the effects of growth. We did not find a superior effect of tDCS in comparison with routine language training and comprehensive training on promoting expressive abilities. This may stem from the S-S method used in this study. The S-S test assesses children’s language development stages by examining significant progress in children’s language development at each stage level to present graded results, which is different from some previous studies (47, 48) that use quantitative assessments of vocabulary understanding and expression and can detect minor improvements more sensitively. The scope and quantity of vocabulary are greatly influenced by factors such as environment, culture, and the level of education of parents, and there are significant differences in the environment, culture, and education levels across various regions of our country. Therefore, there is currently no similar vocabulary scale in China, and thus this study did not use such examination methods. Additionally, we think it may also be related to the intensity and duration of treatment; increasing the duration of the treatment course and follow-up time may reveal differences in the therapeutic effects between various treatment methods.

Previous studies have reported that some children may experience skin itching with tDCS treatment (49). However, this abnormal skin sensation disappears soon after the stimulation is over and does not cause adverse effects to the participants, and this study was consistent with previous studies in this regard. Five children experienced discomfort at the beginning of tDCS treatment, which resolved spontaneously after a few seconds. Therefore, tDCS is a convenient, easy-to-operate, safe, and effective treatment method.

This study had some limitations. The study did not set up a tDCS sham stimulation group, and there was no enough evidence regarding how tDCS interacts with the developing brain and the optimal parameters for pediatric patients. There was no blank control group. For each enrolled child, we gave home guidance based on the evaluation results, and so home training acted as a baseline, that is, this group acted as a control group, and the other three groups were supplemented with other treatments. These preliminary findings can be verified after subsequent studies. Therefore, data from larger samples and a longer follow-up time are needed.

In summary, the combination of tDCS, language-cognitive training, and home training holds great potential on the treatment of children with language delay, which can accelerate the recovery of language and cognitive function and significantly improve communication attitude and basic learning ability. By harnessing the brain’s plasticity and providing targeted cognitive stimulation, this intervention can facilitate effective language learning and contribute to the overall development and well-being of these children.

## Data availability statement

The original contributions presented in the study are included in the article/supplementary material, further inquiries can be directed to the corresponding author.

## Ethics statement

The Research Ethics Committee of the First People’s Hospital of Foshan approved this study (Approval Number: L [2017] No. 6), and written informed consent was obtained from the legal guardian of each participant before enrollment. The studies were conducted in accordance with the local legislation and institutional requirements. Written informed consent for participation in this study was provided by the participants’ legal guardians/next of kin.

## Author contributions

HZ: Conceptualization, Funding acquisition, Writing – review & editing. YX: Writing – original draft, Writing – review & editing, Formal analysis, Methodology. LC: Writing – original draft, Formal analysis, Methodology. JY: Data curation, Writing – original draft, Project administration. ZG: Formal analysis, Writing – original draft, Visualization. PL: Writing – original draft, Project administration.

## References

[1] Sunderajan T Kanhere SV. Speech and language delay in children: prevalence and risk factors. J Family Med Prim Care. (2019) 8:1642–6. doi: 10.4103/jfmpc.jfmpc_162_19, PMID: 31198730 PMC6559061

[2] Zhao S Yao Y Xu J Chen L Huang J Guo T. Prospective cohort study and risk factor analysis of language delay based on outpatient in Xiamen. Chin J Appl Clin Pediatr. (2021) 36:1094–7. doi: 10.3760/cma.j.cn101070-20200214-00151

[3] Simić Klarić A Kolundžić Z Galić S Mejaški BV. Language development in preschool children born after asymmetrical intrauterine growth retardation. Eur J Paediatr Neurol. (2012) 16:132–7. doi: 10.1016/j.ejpn.2011.06.003, PMID: 21764612

[4] Hawa VV Spanoudis G. Toddlers with delayed expressive language: an overview of the characteristics, risk factors and language outcomes. Res Dev Disabil. (2014) 35:400–7. doi: 10.1016/j.ridd.2013.10.027, PMID: 24334229

[5] Law J Levickis P Rodríguez-Ortiz IR Matić A Lyons R Messarra C. Working with the parents and families of children with developmental language disorders: an international perspective. J Commun Disord. (2019) 82:105922. doi: 10.1016/j.jcomdis.2019.105922, PMID: 31425855

[6] Tosh R Arnott W Scarinci N. Parent-implemented home therapy programmes for speech and language: a systematic review. Int J Lang Commun Disord. (2017) 52:253–69. doi: 10.1111/1460-6984.12280, PMID: 27943521

[7] Zhao B Liu Y Liu J Liu Y. Early family intervention in children with language delay: the effect of language level and communication ability. Evid Based Complement Alternat Med. (2022) 2022:3549912. doi: 10.1155/2022/3549912, PMID: 35600939 PMC9122674

[8] Roberts MY Curtis PR Sone BJ Hampton LH. Association of Parent Training with Child Language Development: a systematic review and Meta-analysis. JAMA Pediatr. (2019) 173:671–80. doi: 10.1001/jamapediatrics.2019.1197, PMID: 31107508 PMC6537769

[9] Afshar M Zarifian T Khorrami Banaraki A Noroozi M. Executive functions in Persian-speaking preschool children with speech sound disorders and comparison with their typically developing peers. Appl Neuropsychol Child. (2022) 11:702–12. doi: 10.1080/21622965.2021.1937169, PMID: 34155938

[10] Khan A Yuan K Bao SC Ti CHE Tariq A Anjum N. Can transcranial electrical stimulation facilitate post-stroke cognitive rehabilitation? A systematic review and meta-analysis. Front Rehabil Sci. (2022) 3:795737. doi: 10.3389/fresc.2022.795737, PMID: 36188889 PMC9397778

[11] Qiu A Li X Yang Z Li Z Wang J Yuan H. The effects of combined application of transcranial magnetic stimulation and language training on children with language delay. Minerva Pediatr (Torino). (2023) 75:176–9. doi: 10.23736/S2724-5276.16.04768-X, PMID: 27792211

[12] Chase HW Boudewyn MA Carter CS Phillips ML. Transcranial direct current stimulation: a roadmap for research, from mechanism of action to clinical implementation. Mol Psychiatry. (2020) 25:397–407. doi: 10.1038/s41380-019-0499-9, PMID: 31455860 PMC6981019

[13] Ko MH Yoon JY Jo YJ Son MN Kim DS Kim GW. Home-based transcranial direct current stimulation to enhance cognition in stroke: randomized controlled trial. Stroke. (2022) 53:2992–3001. doi: 10.1161/STROKEAHA.121.037629, PMID: 35975663

[14] De Tommaso B Piedimonte A Caglio MM D'Agata F Campagnoli M Orsi L. The rehabilitative effects on written language of a combined language and parietal dual-tDCS treatment in a stroke case. Neuropsychol Rehabil. (2017) 27:904–18. doi: 10.1080/09602011.2015.1103759, PMID: 26490343

[15] Pisano F Caltagirone C Incoccia C Marangolo P. DUAL-tDCS treatment over the Temporo-parietal cortex enhances writing skills: first evidence from chronic post-stroke aphasia. Life (Basel). (2021) 11:343. doi: 10.3390/life11040343, PMID: 33919714 PMC8070712

[16] Battle DE. Diagnostic and statistical manual of mental disorders (DSM). CoDAS. (2013) 25:190–1. doi: 10.1590/s2317-17822013000200017 24413388

[17] Wan G. Screening and differentiation of the development disorder in speech and language. Chin J Pract Pediatr. (2016) 31:748–51. doi: 10.7504/ek2016100607

[18] Ardito M Botuck S Freeman SE Levy JM. Delivering home-based case management to families with children with mental retardation and developmental disabilities. J Case Manag. (1997) 6:56–61. PMID: 9335725

[19] Blagovechtchenski E Gnedykh D Kurmakaeva D Mkrtychian N Kostromina S Shtyrov Y. Transcranial direct current stimulation (tDCS) of Wernicke's and Broca's areas in studies of language learning and word acquisition. J Vis Exp. (2019):149. doi: 10.3791/59159, PMID: 31355805

[20] Li S. M. (2018). Speech therapy. Beijing, China: Huaxia publishing house.

[21] Yao D Zeng Y Gao M Shen J Zhan J Zhao Z. A research on developmental characteristics of children with language delay in Zhejiang Province, China. Front Pediatr. (2020) 8:479. doi: 10.3389/fped.2020.00479, PMID: 32984202 PMC7477114

[22] Yang Y. M. (2016). Rating scales for children's developmental behavior and mental health. Beijing, China: People’s medical publishing house.

[23] Howse WR Schwartz KL. Knowledge, Skills, Abilities, and Other Characteristics for Remotely Piloted Aircraft Pilots and Operators. AFPC. (2013) 51. doi: 10.21236/ada552499

[24] Liang S Zheng R Zhang L Liu Y Ge K Zhou Z. Effectiveness of parent-training program on children with autism spectrum disorder in China. Int J Dev Disabil. (2020) 68:495–9. doi: 10.1080/20473869.2020.1813063, PMID: 35937175 PMC9351564

[25] Stevens C Fanning J Coch D Sanders L Neville H. Neural mechanisms of selective auditory attention are enhanced by computerized training: electrophysiological evidence from language-impaired and typically developing children. Brain Res. (2008) 1205:55–69. doi: 10.1016/j.brainres.2007.10.108, PMID: 18353284 PMC2426951

[26] Zhang Q Dai X He Y Wei B Dong X Jia H. The application value of transcranial direct current stimulation in treatment of language retardation in children. Hebei Medical Journal. (2020) 42:3. doi: 10.3969/j.issn.1002.7386.2020.03.029

[27] Lee NR Chung SH Song MK Kong YH Joo CU Kim SJ. A comparative analysis of clinical screening test and language specific test in language delay children. Chonnam Med J. (2020) 56:44–9. doi: 10.4068/cmj.2020.56.1.44, PMID: 32021841 PMC6976769

[28] Knudsen HBS Jalali-Moghadam N Nieva S Czaplewska E Laasonen M Gerrits E. Allocation and funding of speech and language therapy for children with developmental language disorders across Europe and beyond. Res Dev Disabil. (2022) 121:104139. doi: 10.1016/j.ridd.2021.104139, PMID: 34979356

[29] Titz C Karbach J. Working memory and executive functions: effects of training on academic achievement. Psychol Res. (2014) 78:852–68. doi: 10.1007/s00426-013-0537-1 24389706

[30] Wiefferink K van Beugen C Wegener Sleeswijk B Gerrits E. Children with language delay referred to Dutch speech and hearing centres: caseload characteristics. Int J Lang Commun Disord. (2020) 55:573–82. doi: 10.1111/1460-6984.12540, PMID: 32459389 PMC7383695

[31] Tang MH Lin CK Lin WH Chen CH Tsai SW Chang YY. The effect of adding a home program to weekly institutional-based therapy for children with undefined developmental delay: a pilot randomized clinical trial. J Chin Med Assoc. (2011) 74:259–66. doi: 10.1016/j.jcma.2011.04.005 21621169

[32] Lin CL Lin CK Yu JJ. The effectiveness of parent participation in occupational therapy for children with developmental delay. Neuropsychiatr Dis Treat. (2018) 14:623–30. doi: 10.2147/NDT.S158688 29503546 PMC5827679

[33] Strauss K Vicari S Valeri G D'Elia L Arima S Fava L. Parent inclusion in early intensive behavioral intervention: the influence of parental stress, parent treatment fidelity and parent-mediated generalization of behavior targets on child outcomes. Res Dev Disabil. (2012) 33:688–703. doi: 10.1016/j.ridd.2011.11.008, PMID: 22188793

[34] Gartstein MA Crawford J Robertson CD. Early markers of language and attention: mutual contributions and the impact of parent-infant interactions. Child Psychiatry Hum Dev. (2008) 39:9–26. doi: 10.1007/s10578-007-0067-4 17570055

[35] Rattaz V Puglisi N Tissot H Favez N. Associations between parent-infant interactions, cortisol and vagal regulation in infants, and socioemotional outcomes: a systematic review. Infant Behav Dev. (2022) 67:101687. doi: 10.1016/j.infbeh.2022.101687, PMID: 35051834

[36] Dong P Xu Q Zhang Y Li D Zhou B Hu C. A multicenter clinical study on parent-implemented early intervention for children with global developmental delay. Front Pediatr. (2023) 11:1052665. doi: 10.3389/fped.2023.1052665, PMID: 36873631 PMC9975705

[37] Friend A Corker S. Enhanced milieu training does not confer additional benefit over standard community interventions for toddlers with language delay. Arch Dis Childhood Educ Pract Edn. (2018) 103:315149. doi: 10.1136/archdischild-2018-315149 29748229

[38] Ferman S Kishon-Rabin L Ganot-Budaga H Karni A. Deficits in explicit language problem solving rather than in implicit learning in specific language impairment: evidence from learning an artificial morphological rule. J Speech Lang Hear Res. (2019) 62:3790–807. doi: 10.1044/2019_JSLHR-L-17-0140, PMID: 31560600

[39] Wu L Song X Ma C Li Y. Effects of early rehabilitation intervention on the children with language retardation. Chinese Scient J Hear Speech Rehabil. (2023) 21:2. doi: 10.3969/j.issn.1672-4933.2023.02.020

[40] Rivera-Urbina GN Nitsche MA Vicario CM Molero-Chamizo A. Applications of transcranial direct current stimulation in children and pediatrics. Rev Neurosci. (2017) 28:173–84. doi: 10.1515/revneuro-2016-0045 27997354

[41] Lefaucheur JP Antal A Ayache SS Benninger DH Brunelin J Cogiamanian F. Evidence-based guidelines on the therapeutic use of transcranial direct current stimulation (tDCS). Clin Neurophysiol. (2017) 128:56–92. doi: 10.1016/j.clinph.2016.10.087, PMID: 27866120

[42] Razza LB da Silva PHR Busatto GF Duran FLS Pereira J De Smet S. Brain perfusion alterations induced by standalone and combined non-invasive brain stimulation over the dorsolateral prefrontal cortex. Biomedicines. (2022) 10:2410. doi: 10.3390/biomedicines10102410, PMID: 36289672 PMC9598449

[43] Baeken C Remue J Vanderhasselt MA Brunoni AR De Witte S Duprat R. Increased left prefrontal brain perfusion after MRI compatible tDCS attenuates momentary ruminative self-referential thoughts. Brain Stimul. (2017) 10:1088–95. doi: 10.1016/j.brs.2017.09.005, PMID: 28917591

[44] Lu H Chan SSM Chan WC Lin C Cheng CPW Linda Chiu Wa L. Randomized controlled trial of TDCS on cognition in 201 seniors with mild neurocognitive disorder. Ann Clin Transl Neurol. (2019) 6:1938–48. doi: 10.1002/acn3.50823, PMID: 31529691 PMC6801176

[45] McLaughlin MR. Speech and language delay in children. Am Fam Physician. (2011) 83:1183–8. PMID: 21568252

[46] Johnson CJ Beitchman JH Young A Escobar M Atkinson L Wilson B. Fourteen-year follow-up of children with and without speech/language impairments: speech/language stability and outcomes. J Speech Lang Hear Res. (1999) 42:744–60. doi: 10.1044/jslhr.4203.744 10391637

[47] Costanzo F Varuzza C Rossi S Sdoia S Varvara P Oliveri M. Reading changes in children and adolescents with dyslexia after transcranial direct current stimulation. Neuroreport. (2016) 27:295–300. doi: 10.1097/WNR.0000000000000536, PMID: 26848997

[48] Richlan F. Developmental dyslexia: dysfunction of a left hemisphere reading network. Front Hum Neurosci. (2012) 6:120. doi: 10.3389/fnhum.2012.00120 22557962 PMC3340948

[49] Andrade AC Magnavita GM Allegro JV Neto CE Lucena Rde C Fregni F. Feasibility of transcranial direct current stimulation use in children aged 5 to 12 years. J Child Neurol. (2014) 29:1360–5. doi: 10.1177/0883073813503710, PMID: 24049057

